# Theoretical Study of Adenine to Guanine Transition Assisted by Water and Formic Acid Using Steered Molecular Dynamic Simulations

**DOI:** 10.3389/fchem.2019.00414

**Published:** 2019-06-12

**Authors:** Santiago Tolosa, Jorge A. Sansón, Antonio Hidalgo

**Affiliations:** Departamento de Ingeniería Química y Química Física, Universidad de Extremadura, Badajoz, Spain

**Keywords:** adenine-guanine transition, SMD simulations, solution reaction mechanisms, genetic mutation, free energy profiles

## Abstract

The free energy profile of the adenine to guanine transition in the gas and aqueous phases was obtained by applying steered molecular dynamic (SMD) simulations. Three processes were considered to explain the mechanism assisted by water and formic acid molecules. The first process is hydrolytic deamination of adenine, then oxidation of the hypoxanthine previously formed, and finally, the animation from xanthine to guanine. In the gas phase these processes indicate a slow and not spontaneous conversion (Δ*G*_g_ = 4.07 kcal·mol^−1^, *k* = 5.59·10^−40^ s^−1^), and a lifetime for guanine of τ = 7.75·10^+22^ s. The presence of solvent makes the transition more difficult by increasing the reaction energy to 26.90 kcal·mol^−1^ and decreasing the speed of the process to 1.63·10^−55^ s^−1^. However, it decreases the energy of the deamination process to −9.63 kcal·mol^−1^ and the lifetime of guanine base to τ = 6.85·10^+17^ s when the surrounding medium used in the transition process is aqueous. The results show that the guanine could participate in genetic mutations based on the lifetimes obtained. Transition states and intermediates structures were analyzed at the molecular dynamic level. This allows to follow the mechanism over time and to calculate thermodynamic and kinetic properties.

## Introduction

Theoretical studies are very helpful to understand aspects related to the chemical and biochemical processes (Warshel, 1991; Müeller et al., 1992; Cramer and Truhlar, 1994; Bała et al., 1996; Náray-Szabó and Warshel, 1997; Tapia and Bertrán, 2002; Ferrario et al., 2006; Kotz et al., 2009). So, the processes that involve the DNA and RNA nucleic acids are frequently studies at molecular level and they are object of many investigations using computational methods (Watson and Crick, 1953).

The interconversion of nitrogenous bases in DNA and RNA in genetic mutations will depend on the medium in which the reaction takes place, the stacking in DNA, and RNA acid strands (Jacquemin et al., 2014; Cerón-Carrasco and Jacquemin, 2015) or the explicit water molecules assisting the mechanism of the process (Cerón-Carrasco et al., 2009a,b,c; Brovarets and Hovorun, 2010; Tolosa et al., 2018a). Also, nucleic acids undergo alterations of their structures from attacks by various agents, like ionizing radiation (Cerón-Carrasco et al., 2010).

Solvation methods (Cramer and Truhlar, 1999; Tomasi et al., 2005; Marenich et al., 2009) are usually employed to consider solvent effects as a continuous medium. Classical molecular dynamic (MD) simulations (Alder and Wainwright, 1959) are a useful method to study the processes at the molecular level, but now the solvent is considered as a discrete medium. The steered molecular dynamic (SMD) technique (Izrailev et al., 1999; Isralewitz et al., 2001) allows us to follow the changes in Gibbs free energy over the course of any elementary process.

**Figure 1:**
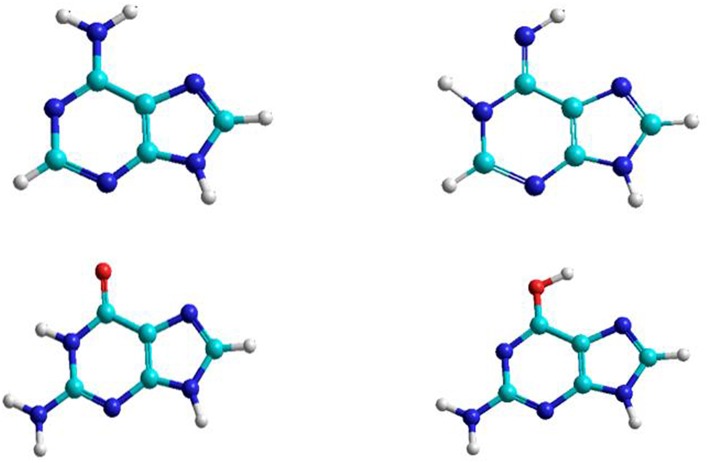
Adenine and guanine tautomeric structures.

The nitrogenous bases: adenine, guanine, cytosine, thymine, and uracil can be associate to form adenine–thymine, guanine–cytosine, and adenine–uracil canonical pairs, although other rare bases may participate in genetic mutation processes (Cerón-Carrasco and Jacquemin, 2015). Adenine and guanine are associated to form the adenine–thymine and guanine-cytosine base pairs. The most stable structures for the adenine base are the amine and imine forms, whereas in guanine there are the keto and enol tautomeric forms (Figure 1). Both bases have been studied in gas and solution phases. The interaction of adenine base with water and oxygen donor molecules has been the subject of studies, because this base can be converted into guanine base and can participate in spontaneous mutation which will be the subject of this work.

**Figure 2:**
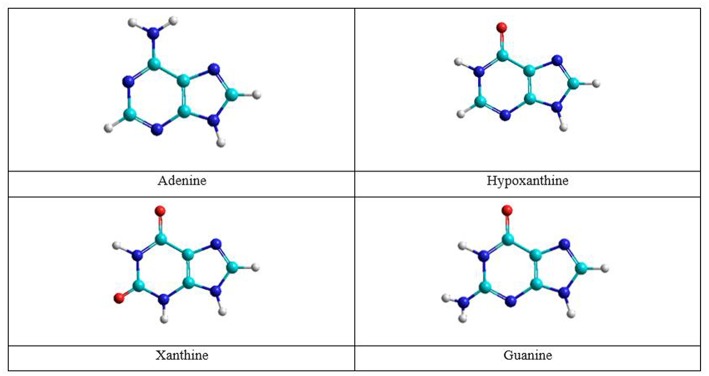
Molecules in the A→G transition process.

As shown in Figure 2 this A→G transition occurs through three processes: first, hydrolytic deamination of adenine base; second, oxidation of the hipoxanthine intermediate; and finally, the animation of the xanthine intermediate to guanine. Specifically, one water molecule attacks the carbon linked to amine group of adenine base to form hypoxanthine and ammonia molecules. Then hipoxanthine oxidation occurs assisted by an oxygen donor molecule (formic acid in this work) to give a xanthine molecule. Finally, the NH_3_ molecule released in the first process attacks by the carbonyl group new to give the guanine base.

## Background

The hydrolytic deamination reaction mechanism of adenine has been theoretically investigated with the DFT method by Zhang et al. (2007), considering the solvent by the PCM model. They found that the deamination may proceed in a stepwise mechanism. A tetrahedral intermediate is formed first by the hydrolysis reaction, and this is followed by the deamination reaction where the C-N link is broken and C = O group is formed. The study shows that the deamination with one water molecule does not take place because of a higher barrier. However, it may be possible if several water molecules are considered, because one molecule can act as a catalyst and the others act as assistant molecules. This is agreement with the experimental results.

In other similar work, the adenine deamination was studied by Zhu and Meng (2009) by four different mechanisms using the DFT method. The most favorable pathway describes a tetrahedral intermediate formed through a hydrolysis reaction on the C-N bond, then the deamination reaction takes place breaking the C-N bond and forming a C = O bond. The activation barriers are high, and it is difficult for this process to happen with such high activation barriers.

Later, Zheng and Meng (2009) studied the hydrolytic deamination mechanism of adenine using several water-assistant molecules and the density functional method at the DFT method. When the first water molecule attack to adenine base a tetrahedral intermediate is formed. Then, two different intermediates were studied. In A-pathway, the second water molecule acts as a bridge to transfer the hydrogen atom. In B-pathway, the second water molecule is not involved in the formation of the transition structure and only acts as a aqueous medium. Energy barriers of the two processes are 23.40 and 37.17 kcal·mol^−1^, respectively.

Deamination of protonated adenine to produce hypoxanthine has also been studied by Wang and Meng (2010) in acid medium and assisted with a water molecule, using the DFT method. Because adenine could be protonated on different nitrogen atoms, four pathways were investigated. The pathway where the nitrogen closer to the NH_2_ amine group is protonated presents the lowest energy barrier of 28.9 kcal·mol^−1^.The first step and rate-determining is the nucleophilic attack of water molecule to form a tetrahedral structure. The results show that adenine deamination under acidic medium is more favorable than in neutral conditions.

Alrawashdeh et al. (2013) studied several mechanisms for the deamination of adenine with up to four H_2_O molecules in basic medium and at different levels of theory. The effect of aqueous solvent was computed using the PCM model. Deamination with a water molecule presents a high activation barrier (190 kJ mol^−1^). The addition of 1 s water molecule reduces this barrier by 68 kJ mol^−1^. The most favorable mechanism with three water molecules presents an activation energy of 139 (in gas phase) and 137 kJ mol^−1^ (in solution phases).

The oxidation mechanism of hypoxanthine has been investigated by Tafasse (2015). This theoretical study aimed to predict the transition state structure in the mechanism of oxidation with xanthine oxidase (XO) enzyme. The DFT/B3LYP method was used in the geometry optimization and it revealed that oxidation of hypoxanthine by xanthine oxidase follows a stepwise mechanism: (a) abstraction of a proton by Glu1226 from the hydroxyl hydrogen of XO followed by nucleophilic attack on the carbon of the substrate, and (b) then a proton transfer from substrate to enzyme to produce the hypoxanthine linked to the enzyme complex. The process was exothermic and with a high energy barrier.

The catalytic mechanism of guanine amination was investigated by a combination of QM calculations using the ONIOM method and MD simulations (Yao et al., 2007). The process was assisted by a Zn-metalloenzyme and two residues (Glutamate 55 and Aspartate 114). The proposed mechanism is initiated by a proton transfer from a Zn-bound water molecule to protonate Asp114. The protonated Asp114 can perform the proton transfer to the guanine, facilitating the nucleophilic attack on the nitrogenous base. The residue Glu55 then perform the proton transfer from the Zn-hydroxide to the amino group of the intermediate. The C-NH_3_ bond breaks forming ammonia which leaves the active site and xanthine is freed with a barrier about 8 kcal·mol^−1^.

The deamination of guanine with H_2_O and OH^−^ has been realized with *ab initio* calculations by Uddin et al. (2011). Optimized geometries of all species were determined at different levels of theory and the Gibbs free energies were also determined. Deamination with OH^−^ radical was found to have an activation barrier of 155 kJ mol^−1^ compared to 187 kJ mol^−1^ for the reaction with H_2_O. The lowest overall activation energy, 144 kJ mol^−1^, was obtained using the H_2_O/OH^−^ pair.

No theoretical studies have been realized for this A→G transition, although the separate processes of deamination, oxidation, and animation have been performed with model systems above mentioned. Here, we present the first study that uses the SMD technique to calculate molecular properties of this A→G process.

## Methodology

The formalism used in this work has been the same as the one used in previous studies by our research group, so for more detailed information it can be consulted in the work of cytosine to thymine conversion (Tolosa et al., 2019). Here we will only mention the methods used and some specific considerations of the simulated system.

The LJ (12-6-1) potential was used to describe the solute-solvent interaction, where van der Waals parameters are taken from the AMBER force field (Cornell et al., 1993) and the charge on each solute are the initially obtained by Mulliken method (Mulliken, 1955) and, subsequently, recalculated with the RESP method (Damm et al., 1997). The solvent charges were the TIP3P charges (Jorgensen and Tirado-Rives, 1988; Kaminski et al., 1994; Jorgensen et al., 1996).

The initial geometry of the Adenine-Water-Formic complex (denoted hereafter as A-W-F system), with the formic acid and water reactants about 2.0 Å from adenine, was obtained with the AMBER12 software (Case et al., 2012) at 298.15 K.

The reaction coordinates used to carry out the SMD simulations can be expressed as:

**Table:** 

$\begin{array}{l} \text{R}{\text{C}}_{1\text{S}1\text{A}}=\text{d}\left(O_{21}-{\text{C}}_{5}\right), \end{array}$	$\begin{array}{l} \text{R}{\text{C}}_{2\text{S}1\text{A}}=\text{d}\left({\text{H}}_{23}-\;{\text{N}}_{7}\right)-\text{d}\left({\text{H}}_{23}-{\text{O}}_{21}\right) \end{array}$
$\begin{array}{l} \text{R}{\text{C}}_{1\text{S}2\text{A}}=\text{d}\left({\text{H}}_{22}-{\text{N}}_{10}\right)-\text{d}\left({\text{H}}_{22}-{\text{O}}_{21}\right), \end{array}$	$\begin{array}{l} \text{R}{\text{C}}_{2\text{S}2\text{A}}=\text{d}\left({\text{C}}_{6}-{\text{N}}_{7}\right) \end{array}$
$\begin{array}{l} \text{R}{\text{C}}_{1\text{S}1\text{B}}=\text{d}\left({\text{H}}_{22}-{\text{N}}_{10}\right), \end{array}$	$\begin{array}{l} \text{R}{\text{C}}_{2\text{S}1\text{B}}=\text{d}\left({\text{O}}_{21}-{\text{C}}_{6}\right) \end{array}$
$\begin{array}{l} \text{R}{\text{C}}_{1\text{S}2\text{B}}=\text{d}\left({\text{H}}_{23}-{\text{N}}_{7}\right)-\text{d}\left({\text{H}}_{23}-{\text{O}}_{21}\right), \end{array}$	$\begin{array}{l} \text{R}{\text{C}}_{2\text{S}2\text{B}}=\text{d}\left({\text{C}}_{6}-{\text{N}}_{7}\right) \end{array}$
$\begin{array}{l} \text{R}{\text{C}}_{1\text{S}3\text{C}}=\text{d}\left({\text{O}}_{18}-{\text{C}}_{11}\right), \end{array}$	$\begin{array}{l} \text{R}{\text{C}}_{2\text{S}3\text{C}}=\text{d}\left({\text{H}}_{20}-{\text{N}}_{13}\right)-\text{d}\left({\text{H}}_{20}-{\text{O}}_{18}\right) \end{array}$
$\begin{array}{l} \text{R}{\text{C}}_{1\text{S}4\text{C}}=\text{d}\left({\text{H}}_{12}-{\text{C}}_{16}\right)-\text{d}\left({\text{H}}_{12}-{\text{C}}_{11}\right) \end{array}$	
$\begin{array}{l} \text{R}{\text{C}}_{1\text{S}5\text{D}}=\text{d}\left({\text{N}}_{7}-{\text{C}}_{11}\right), \end{array}$	$\begin{array}{l} \text{R}{\text{C}}_{2\text{S}5\text{D}}=\;\text{d}\left({\text{H}}_{22}-{\text{O}}_{18}\right)-\text{d}\left(H_{22}-{\text{N}}_{7}\right) \end{array}$
$\begin{array}{l} \text{R}{\text{C}}_{1\text{S}6\text{D}}=\text{d}\left({\text{H}}_{20}-{\text{O}}_{18}\right)-\;\text{d}\left({\text{H}}_{20}-{\text{N}}_{13}\right) \end{array}$	
$\begin{array}{l} \text{R}{\text{C}}_{1\text{S}5\text{E}}=\text{d}\left({\text{H}}_{20}-{\text{O}}_{18}\right)-\;\text{d}\left({\text{H}}_{20}-{\text{N}}_{13}\right) \end{array}$	
$\begin{array}{l} \text{R}{\text{C}}_{1\text{S}6\text{E}}=\text{d}\left({\text{N}}_{7}-{\text{C}}_{11}\right), \end{array}$	$\begin{array}{l} \text{R}{\text{C}}_{1\text{S}6\text{E}}=\text{d}\left({\text{H}}_{22}-{\text{O}}_{18}\right)-\text{d}\left({\text{H}}_{22}-{\text{N}}_{7}\right). \end{array}$

Long-range electrostatic interactions were considered by the Ewald summation (Ewald, 1921) and the Jarzynski's equality (Jarzynski, 1997) was used to calculate Gibbs energy differences between two equilibrium states. The activation and reaction energies were determined through the evolution of the process, as has been shown in several works (Tolosa et al., 2014, 2016, 2017a,b, 2018a,b; Tolosa et al., 2019).

Simulations were performed with the QM/MM method and the semi-empirical Hamiltonian AM1 method (Dewar et al., 1985). The system was partitioned, applying quantum calculations to the A-W-F (QM subsystem) an a classical way to the H_2_O solvent (MM subsystem). HCOOH and H_2_O molecules that assisted proton transfer were included in the QM part.

## Results

### Processes

**Figure 3:**
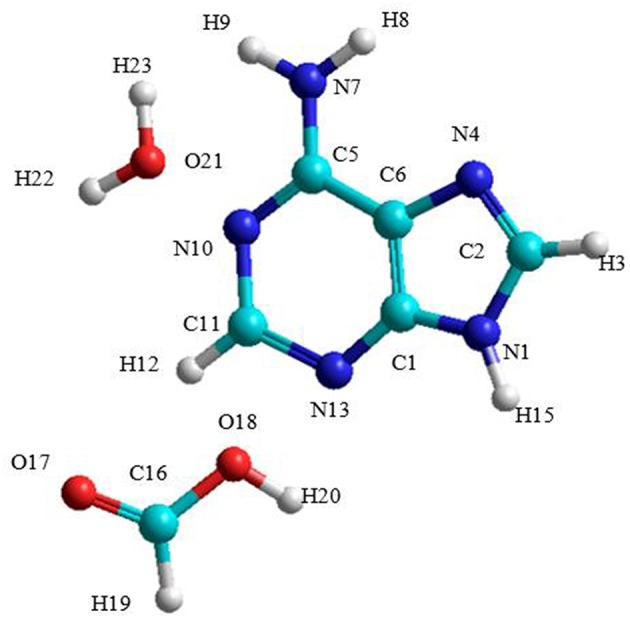
Structure of the A-W-F system.

The A→G transition was studied using SMD simulations for each process. The initial structure of the A-W-F system is shown in Figure 3. The initial configuration between water and formic acid assistant molecules and adenine base is that where these molecules were oriented and positioned to look for the ideal situation to start the first step of the mechanism. The initial A-W-F geometry are reported in Table S1.

**Figure 4:**
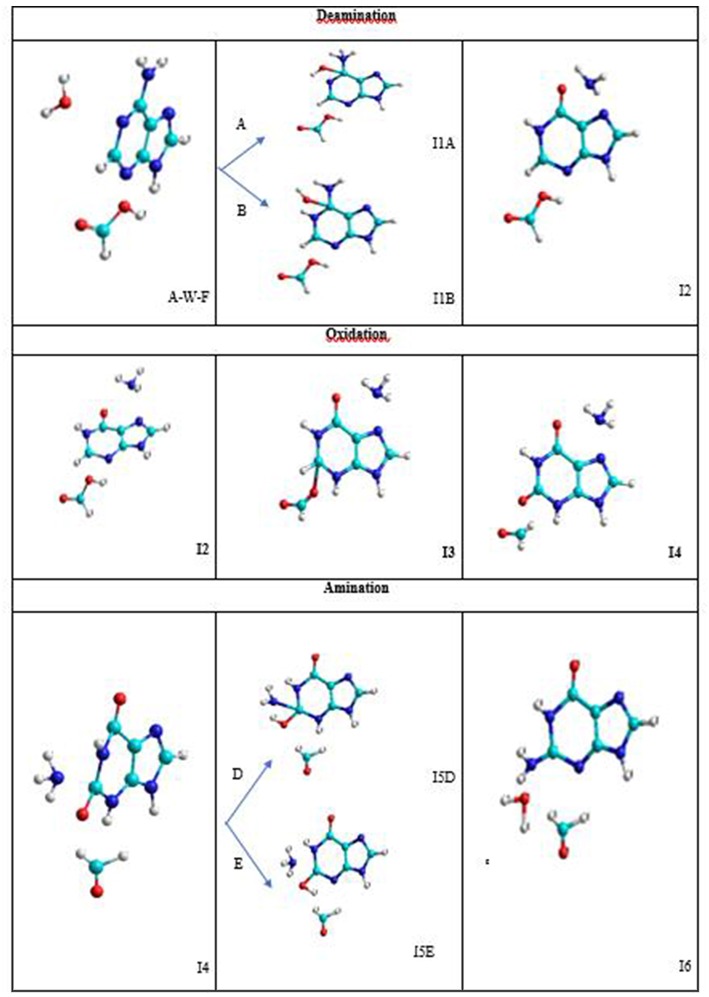
Intermediate structures in the A→G transition.

The conversion mechanism considered in this work is described in three processes. The intermediates and transition structures are schematized in Figure 4 and their Cartesian coordinates, obtained from SMD simulations, are given in Table S2. In the first process, the hydrolytic deamination of adenine to hypoxanthine is performed. The second process is the oxidation of hypoxanthine to xanthine, and the third is the amination of xanthine to guanine base. All of these three processes are described by stepwise mechanisms. The I2, I4, and I6 final structures in each process show separate molecules (about 4Å) avoiding the representation of a configuration where the assistant molecules and the base can be associated by some type of bond.

In the deamination process, the H_2_O molecule attacks the C5 atom in a plane perpendicular to the adenine base and forms an I1 intermediate with an OH hydroxyl group linked to this carbon. The other hydrogen of the water molecule is transferred to the amine nitrogen N7 (A-pathway) or to the imine nitrogen N10 (B-pathway). In the next step, an intramolecular proton transfer from the OH group to the N10 nitrogen (A-pathway) or to the N7 (B-pathway) is performed. This elongates the C5-N7 bond before it ruptures and forms the hypoxanthine and ammonia molecules (I2 system).

In the oxidation process, the formic acid attacks in the same plane to the hypoxanthine molecule by the C11 carbon atom. Specifically, the acid protonates the nitrogen N13 and remains as the HCOO^−^ anion that stimulates its nucleophilic attack on C11 atom forming the I3 intermediate. Then, the hydrogen H12 bonded to the C11 atom is transferred to the C16 carbon of the acid and the C16-O18 bond is broken forming the corresponding aldehyde HCOH (I4 system).

In the amination process, the ammonia molecule formed in the deamination process attacks perpendicular to the base by C11 carbon atom of the xanthine with the proton transfer to the O18 oxygen from the N7 nitrogen (D-pathway) or N13 nitrogen (E-pathway) to form the I5 intermediate. In a second step, the proton transfer from N13 nitrogen to the hydroxyl oxygen (D-pathway) or N7 nitrogen (E-pathway) is performed, releasing a water molecule once the C11-O18 bond is broken (I6 system).

### Structures and Energies From SMD Simulations

The transition states and intermediates states along the A→G transition have been visualized previously in Figure 4 and its Cartesian coordinates are given in Table S2. The activation (Δ*G*^‡^) and reaction (Δ*G*) energies, forward rate constants (k_*f*_), equilibrium constants (K), and intermediate lifetimes (τ), from SMD simulations in both phases, are presented in Table 1 (gas phase) and Table 2 (solution phase) and compared with other studies in Table 3.

**Table 1:** Properties for A→G transition in the gas phase.

	**Pathway**	**Δ *G*^‡^^,^^a^**	**Δ *G*^b^**	***K*^c^**	**$K_{\text{f}}^{\text{d}}$**	***τ^e^***
Deamination	1A-step	61.11	21.72	2.82·10^−16^	9.37·10^−33^	1.26·10^+16^
	2A-step	47.72	−27.48	1.43·10^+20^	1.44·10^−22^	2.30·10^+42^
	A-process	71.05	−5.76	1.68·10^+04^	4.80·10^−40^	2.30·10^+42^
	1B-step	59.60	9.50	1.08·10^−07^	2.79·10^−31^	7.70·10^+23^
	2B-step	54.45	−8.27	1.16·10^+06^	6.07·10^−28^	8.76·10^+33^
	B-process	63.95	0.67	6.78·10^−01^	7.74·10^−35^	8.76·10^+33^
Oxidation	3C-step	53.90	4.61	4.16·10^−04^	1.82·10^−27^	2.28·10^+33^
	4C-step	75.30	−2.79	1.11·10^+02^	3.76·10^−43^	3.03·10^+44^
	C-process	79.91	1.82	4.63·10^−02^	1.52·10^−46^	3.03·10^+44^
Amination	5D-Step	42.51	−1.69	1.74·10^+01^	4.11·10^−19^	4.22·10^+19^
	6D-Step	53.46	18.67	2.03·10^−14^	3.83·10^−27^	5.29·10^+12^
	D-process	51.77	16.98	3.52·10^−13^	6.66·10^−26^	5.29·10^+12^
	5E-step	54.69	4.54	4.68·10^−04^	4.79·10^−28^	9.60·10^+23^
	6E-step	52.52	3.87	1.45·10^−03^	8.76·10^−30^	7.75·10^+22^
	E-process	57.06	8.41	6.79·10^−07^	8.76·10^−30^	7.75·10^+22^
Global	A→G^f^	74.15	4.07	1.03·10^−03^	2.56·10^−42^	4.05·10^+38^

a *Activation energy (in kcal·mol^−1^)*.

b *Reaction energy (in kcal·mol^−1^)*.

c *Equilibrium constant evaluated as* $K=e^{-\;\frac{\Delta G}{RT}}$.

d *Forward rate constant* (in s^−1^) evaluated as kf=BKThe−  ΔG∓RT.

e *Lifetime (in s) of the final species in each step evaluated from the energy barrier of the inverse process as* $\tau =\frac{\text{1}}{k_{r}}$.

f *Values considering the A-C-E pathway*.

‡ *Activation energy*.

**Table 2:** Properties for A→G transition in solution phase.

	**Pathway**	**Δ *G*^‡^^,^^a^**	**Δ *G*^b^**	***K*^c^**	**$k_{\text{f}}^{\text{d}}$**	***τ^e^***
Deamination	1A-step	59.31	17.31	2.01·10^−13^	1.96·10^−31^	1.03·10^+18^
	2A-step	50.74	−26.86	5.02·10^+19^	3.78·10^−25^	1.35·10^+44^
	A-process	68.05	−9.63	1.16·10^+07^	7.62·10^−38^	1.35·10^+44^
	1B-step	55.25	4.90	2.55·10^−04^	1.86·10^−28^	1.37·10^+24^
	2B-step	55.30	−10.68	6.81·10^+07^	1.71·10^−28^	3.98·10^+35^
	B-process	60.30	−5.78	1.74·10^+04^	3.68·10^−32^	3.98·10^+35^
Oxidation	3-step	56.30	3.10	5.32·10^−03^	3.16·10^−29^	1.68·10^+26^
	4-step	79.41	8.85	3.23·10^−07^	3.55·10^−46^	9.10·10^+38^
	C-process	82.51	11.95	1.72·10^−09^	1.89·10^−48^	9.10·10^+38^
Amination	5D-Step	48.61	7.73	2.14·10^−06^	1.38·10^−23^	1.55·10^+17^
	6D-Step	54.09	29.43	2.60·10^−22^	1.32·10^−27^	1.87·10^+05^
	D-process	61.82	37.16	5.56·10^−28^	2.82·10^−33^	1.87·10^+05^
	5E-Step	57.88	8.81	3.45·10^−07^	2.19310^−30^	1.58·10^+23^
	6E-Step	59.54	15.78	9.10·10^−14^	1.33·10^−31^	6.85·10^+17^
	E-process	68.35	24.59	9.21·10^−19^	2.13·10^−34^	6.85·10^+17^
Global	A→G^f^	72.88	26.90	1.86·10^−20^	2.18·10^−41^	8.5310^+20^

a *Activation energy (in kcal·mol^−1^)*.

b *Reaction energy (in kcal·mol^−1^)*.

c *Equilibrium constant evaluated as* $K=e^{-\;\frac{\Delta G}{RT}}$.

d *Forward rate constant (in s^−1^) evaluated as*kf=BKThe−  ΔG∓RT.

e *Lifetime (in s) of the final species in each step evaluated from the energy barrier of the inverse process as* $\tau =\frac{\text{1}}{{\text{k}}_{\text{r}}}$.

f *Values considering the A-C-E pathway*.

‡ *Activation energy*.

**Table 3:** Activation and reaction energies (in kcal·mol^−1^).

**Processes**	**Δ*G*^‡^**	**Δ*G***
**Deamination**		
DFTB/B3LYP/PCM^a^^,^^b^	62.40^a^, 64.04^b^	−1.2^a^, −1.18^b^
SMD-Gas (A-pathway)	71.05	−5.76
SMD-Solution (A-pathway)	68.05	−9.63
**Oxidation**		
SMD- Gas (C-pathway)	79.91	1.82
SMD-Solution (C-pathway)	82.51	11.95
**Amination**		
DFTB/B3LYP/6.31G(d)^c^	65.47	3.03
SMD- Gas (E-pathway)	57.06	8.41
SMD-Solution (E-pathway)	68.35	24.59
**A→G Transition**		
SMD- Gas (A-C-E-pathway)	74.15	4.07
SMD-Solution (A-C-E-pathway)	72.88	26.90

a *Values obtained by Zhang et al. (2007) using similar pathway but with some differences*.

b *Values obtained by Zhu and Meng (2009)*.

c *Values obtained by Uddin et al. (2011)*.

‡ *Activation energy*.

#### Gas Phase Simulations

In the deamination process via A-pathway, the activation energies were as follows: ΔG_*TS*1*A*_^‡^ = 61.11 kcal·mol^−1^ and ΔG_*TS*2*A*_^‡^ = 47.72 kcal·mol^−1^ for the first two steps, respect to the initial system of each step. The deamination was slightly exergonic, ΔG_*A*_ = −5.76 kcal·mol^−1^. When the process follows the B-pathway, the first step is less endergonic with respect to the A-pathway but not so the second step. The process now is fastest (ΔG_*B*_^‡^ = 63.95 kcal·mol^−1^; k_*B*_ = 7.74·10^−35^ s^−1^) and slightly endergonic (ΔG_*B*_ = 0.67 kcal·mol^−1^). Although both processes have very similar energy profiles, the most thermodynamically favorable is the A-pathway, because the intramolecular proton transfer observed is more exergonic when it is performed on the N10 imine nitrogen (A2-step) rather than on the N7 amine nitrogen (B2-step).

The barrier energy observed when the water molecule attacks the adenine base is very high showing that the formation of the intermediate I1A is the rate-determining steps of this deamination process, with rate constant k_1*A*_ = 9.37·10^−33^ s^−1^. The protonation step on the N10 atom in structure I1A is the most exergonic step of the deamination (and of the A→G global transformation) with energy of Δ*G*_I2A_ = −27.48 kcal·mol^−1^. The stability and the long lifetime of the hypoxanthine molecule via A-pathway (τ_2A_ = 2.30·10^+42^ s^1^) and the existing equilibrium between cytosine and hypoxanthine molecules via B-pathway (K_B_ = 6.78·10^−01^) should also be highlighted.

Examining the stationary state structures (see Table S2), we can see that the transition states are formed when the distance d_O21−C6_ is 1.65 Å, and when the distances d_H23−N7_ (TS1A) and d_H22−N10_ (TS1B) are 1.22 and 1.37 Å, respectively. In I1A and I1B intermediates the distance d_O21−C6_ decreases to 1.48 Å whereas the C6-N7 bond was elongated to 1.7 Å in the I1A structure. In the second step, the TS2A structure is formed when the H23 atom is 1.48 Å from the O21 donor oxygen and 1.33 Å from the N10 acceptor nitrogen, whereas TS2B is formed when the H22 hydrogen is 1.28 Å from the O21 donor oxygen and 1.29 Å from the N7 acceptor nitrogen. The process ends when the hypoxanthine and ammonia molecules are far away (d_C6_-_N7_ = 4.0 Å).

In the oxidation process, the activation energies were as follows: Δ G_*TS*3*C*_^‡^ = 53.90 kcal·mol^−1^ and ΔG_*TS*4*C*_^‡^ = 75.30 kcal·mol^−1^. The process was slightly endergonic, with a global reaction energy of ΔG_C_ = 1.82 kcal·mol^−1^, and very slow with a value of k_*C*_ = 1.52·10^−46^·s^−1^. Although the 3C-step (describing simultaneously a nucleophilic attack and a protonation) was slow, the highest activation barrier is the rupture of the C16-O18 bond that links the formic acid with the hypoxanthine molecule (ΔG_*TS*4*C*_^‡^ = 75.30 kcal·mol^−1^) making it the rate-determining step of the oxidation s and of the A→G global transformation. However, the formation of a stable molecule such as xanthine (τ_4C_ = 3.03·10^+44^ s) justifies the exergonic character of this step (ΔG_4C_ = −2.79 kcal·mol^−1^) even though the global process (ΔG_C_ = 1.82 kcal·mol^−1^).

The stationary state structures reveals that in the TS3 state, the formic acid and hypoxanthine molecules are in perpendicular planes and that the O18 oxygen is located 1.72 and 1.38 Å from the acceptor and donor carbon atoms, respectively; while the H20 hydrogen is at 1.30 Å from the N13 and O18 atoms. In the I3 intermediate, these distances were 1.42 and 1.02 Å with respect to their acceptor atoms. The structure of TS4 shows a four-membered ring where the H12 and O18 atoms involved in the transfers are at 1.31 and 1.56 Å from their acceptor atoms. In the I4 final structure, the C = O bond distance was 1.27 Å and the formaldehyde and xanthine molecules were separated by 4.0 Å.

In the amination process, the activation energies were: ΔG_*TS*5*D*_^‡^ = 42.51 kcal·mol^−1^ and ΔG_*TS*6*D*_^‡^ = 53.46 kcal·mol^−1^. The process was endergonic (Δ*G*_D_ = 16.98 kcal·mol^−1^) and slow (k_*D*_ = 6.66·10^−26^·s^−1^). The nucleophilic attack of the ammonia molecule on the C11 atom and the protonation of O18 oxygen (5D-step) present a barrier lower that the ones that describe the proton transfer from N13 to O18 oxygen and the break of the O18-C11 bond (6D-step). The guanine molecule presents a lifetime shorter than that of the hypoxanthine and xanthine intermediates (τ_D_ = 5.29·10^+12^ s) and the equilibrium is clearly shifted to xanthine molecule (K_D_ = 6.66·10^−26^).

When the amination process begins with the intramolecular proton transfer from N13 to O18 atoms (E-pathway), the process turns out to be slower (*k*_E_ = 8.76·10^−30^ s^−1^) but less endergonic (Δ*G*_E_ = 8.41 kcal·mol^−1^) and the lifetime of guanine increases considerably to τ_E_ = 7.75·10^+22^ s. Hence, the E-pathway is considered to be the most thermodynamically favorable. Namely, the second step of this process where the nucleophilic attack of the NH_3_ molecule releases a water molecule (6E-step) needs less energy than the intramolecular proton transfer (6D-step).

The N7 nitrogen of the ammonia molecule approaches at 1.76 Å in the TS5 state and at 1.50 Å in the I5 intermediate with respect to the C11 atom, while the O18 oxygen goes from 1.36 Å in the TS5 state to 1.42 Å in the I5 intermediate. The H22 hydrogen transferred from the ammonia to the xanthine molecules is at 1.41 Å with respect to the N7 donor atom and at 1.26 Å with respect to the O18 acceptor in the TS5 state. In the second step of this amination process, a TS6 structure is obtained where the H20 hydrogen transferred is at 1.30 Å from the N13 donor atom and at 1.32 Å from the O18 acceptor atom, elongating the distance O18-C11 to 1.47 Å before breaking and separating the water molecule up to 4.0 Å.

For the E-pathway, the H20 atom is at 1.31 Å from the O18 atom and at 1.54 Å from the N13 nitrogen in the TS5 state. The H22 atom is at 1.28 Å and 1.26 Å from the O18 and N7 atoms, respectively, in the TS6 structure, and the distance d_N7−C11_ is 1.61 Å. The final state presents the water molecule at almost 4.0 Å from the guanine base and the N7 atom remains linked to the C11 at a distance of 1.40 Å.

**Figure 5:**
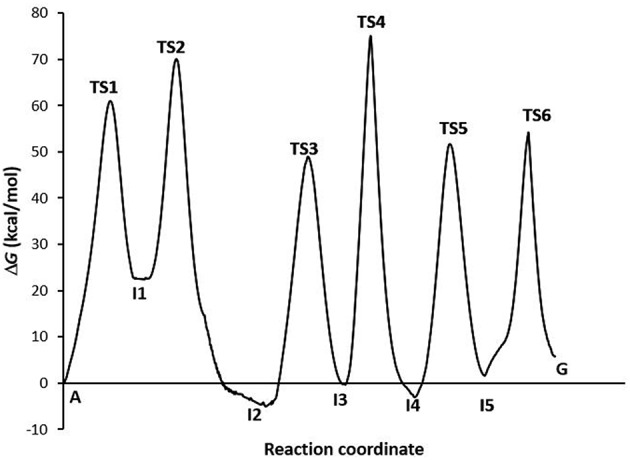
Profiles via A-C-E processes in the gas phase.

The profile of the deamination, oxidation, and amination processes (Figure 5) shows that the determining step is the formation of the TS4 transition state (Δ*G*^‡^ = 74.15 kcal·mol^−1^, *k* = 2.56·10^−42^). The guanine product exists in a slightly higher energy state than the other stable species of each process (adenine, hypoxanthine, and xanthine), what shows the slight endergonic character of the A→G transition (Δ*G* = 4.07 kcal·mol^−1^.Wwe can conclude that the A→G transition process in the gas phase is thermodynamically and kinetically unfavorable, although the guanine base could participate in genetic mutations (τ = 4.05·10^+38^ s) and an equilibrium between both bases is observed (*K* = 1.03·10^−03^).

#### Solution Phase Simulation

Examining the stationary state structures, we can see that they are similar to those of the gas phase simulations. So, we will avoid repeating this analysis and show their Cartesian coordinates in Table S2 of the supplementary information, and we will focus only on the energy analysis.

Now the deamination process via A-pathway presents activation energies of ΔG_*TS*1*A*_^‡^ = 59.31 kcal·mol^−1^ and ΔG_*TS*2*A*_^‡^ = 50.74 kcal·mol^−1^ and reaction energies of ΔG_1*A*_ = 17.31 kcal·mol^−1^ and ΔG_2*A*_ = −26.86 kcal·mol^−1^. When the process follows the B-pathway, the global result is more favorable kinetically but not thermodynamically (ΔG_*B*_^‡^ = 60.30 and ΔG_*B*_ = −5.78 kcal·mol^−1^). The activation energy of the nucleophilic attack on adenine is the rate-determining step of this deamination process (k_1*A*_ = 1.96·10^−31^s^−1^ and k_1*B*_ = 1.86·10^−28^·s^−1^), while the protonation of the N10 atom in the I1A intermediate is the step that is more exergonic (Δ*G*_2A_ = −26.86 kcal·mol^−1^). Likewise, the long lifetime of hypoxanthine (τ_2A_ = 1.35·10^+44^·s, τ_2B_ = 3.98·10^+35^ s^−^) shows the stability of this molecule.

In the oxidation process, the activation energies were as follows: ΔG_*TS*3*C*_^‡^ = 56.30 kcal·mol^−1^ and ΔG_*TS*4*C*_^‡^ = 79.41 kcal·mol^−1^. The process was endergonic, with a total reaction energy ΔG_C_ = 11.95 kcal·mol^−1^, and very slow (k_*C*_ = 1.89·10^−48^ s^−1^). The highest activation barrier necessary for the break of the C16-O18 link and the formation of the C16-H12 link between both molecules makes it the rate-determining step of the oxidation and of the total conversion. The xanthine molecule with a lifetime of τ_4_ = 9.10·10^+38^s turned out to be a stable species.

In the amination process, the activation energies were ΔG_*TS*5*D*_^‡^ = 48.61 kcal·mol^−1^ and ΔG_*TS*6*D*_^‡^ = 54.09 kcal·mol^−1^. The process was very endergonic with a total reaction energy Δ*G*_D_ = 37.16 kcal·mol^−1^ and with a constant k_D_ = 2.82·10^−33^ s^−1^. The break of the C11-O18 present a barrier higher than that which describes the nucleophilic attack of NH3 molecule on C11 atom, the formation of guanine molecule had a lifetime less than that of the other species (τ = 1.87·10^+5^s) and the equilibrium is shifted to xanthine molecule (K_D_ = 5.56·10^−28^).

When the animation is followed by E-pathway, the process becomes slower (Δ$G_{\text{E}}^{\text{z}}$ = 68.35 kcal·mol^−1^) and less endergonic (Δ*G*_E_ = 24.59 kcal·mol^−1^), increasing the lifetime of guanine base to τ_E_ = 6.85·10^+17^ s. These changes can be justified in the same way as in the gas phase simulation, but now the presence of the solvent makes proton transfer from N10 to O18 atoms (6E-step) less favorable and the process becomes more endergonic.

**Figure 6:**
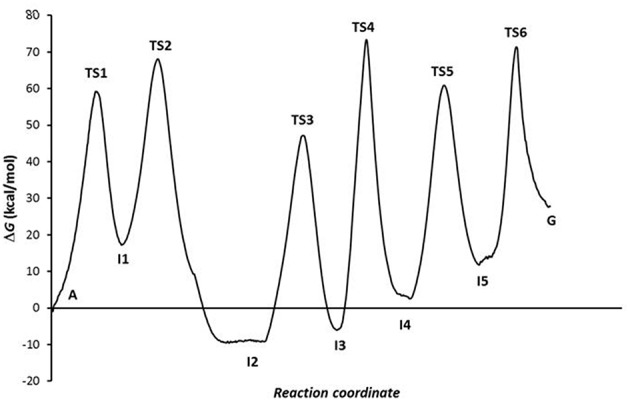
Profiles via A-C-E processes in the solution phase.

The combination of the three processes presents an energy profile (Figure 6 shows the A-C-E pathway) being the TS4 structure the rate-determining step as the guanine product exists in a higher energy state than the adenine, hypoxanthine, and xanthine species. We can conclude that the process of A→G process in solution is shifted to adenine base (*K* = 1.86·10^−20^) and is thermodynamic (Δ*G* = 26.90 kcal·mol^−1^) and kinetically (*k* = 2.18·10^−41^ s^−1^) unfavorable, where the guanine base could participate in genetic mutation processes (τ = 8.53·10^+20^ s).

Our results differ slightly from those obtained by other studies (Yao et al., 2007; Zhang et al., 2007; Zheng and Meng, 2009; Zhu and Meng, 2009; Wang and Meng, 2010; Uddin et al., 2011; Alrawashdeh et al., 2013; Tafasse, 2015), showing processes with high barriers and low reaction energies. In deamination and animation processes, this is due to the level of calculation used in these studies (usually from electronic structure calculations) and to the different mechanisms employed. For the oxidation process the difference, apart from the level of calculation used, is in the models used with enzymes such as oxidase XO, as previously described. So, the comparison of results loses meaning and will not be done.

From the comparison between the different processes, it can be observed that the most favorable energetically is deamination, while amination is the one that marks the endergonic character, and oxidation justifies the slowness of the A→G transition. It can also be seen from the results presented in Table 3 that the route A-C-E has the lowest energy cost.

**Figure 7:**
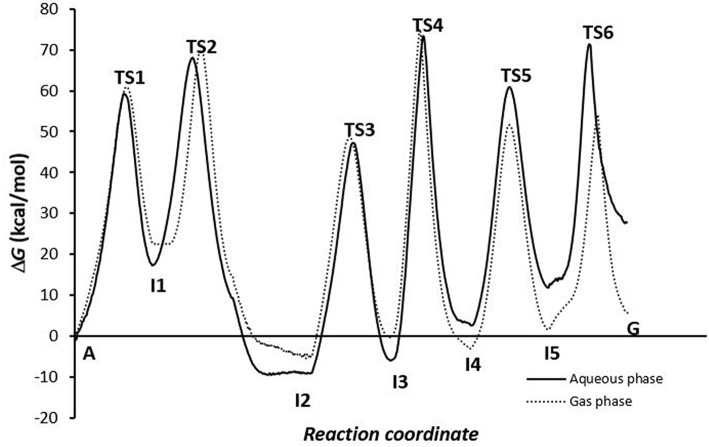
Profiles for the conversion via A-C-E pathway.

Of the energy profiles of A→G transition according to the phase in which the simulation was carried out (Figure 7), we can see that this effect is not very important for the activation and reaction energies of deamination and oxidation processes, but meaning in amination process. It is more favorable when the global processes are simulated in the gas phase. In solution, the assistant molecules and the adenine base may be surrounded by aqueous solvent molecules, which makes proton transfer more difficult and increases the energies necessary for these processes to occur. However, at other times (as in the deamination process) some of the molecules of the medium can facilitate the process by participating as a second molecule in assisting the mechanism.

## Conclusions

In the A→G transition none of the simulated processes were both thermodynamically and kinetically favorable, except for the deamination which was slightly exergonic. The result, considering all processes, leads us to conclude that the A→G transition is not spontaneous, although somewhat more favorable in vacuum. Also, the lifetimes of guanine show that this base could participate in genetic mutation processes.

The reaction profiles are similar for both phases, although the final result shows lower energies when the transition is simulated in gas phase, justified by the amination process. The effect of the medium can influence the mechanism that is followed depending on whether there are molecules surrounding the system that hinder or favor the transfer, as happens for the deamination (more exergonic in solution) or oxidation and amination (more endergonic in solution) processes.

Transition states that describe nucleophilic attacks present high barriers. The highest the barrier to overcome is the TS4 structure that describes the break between the base and the formic acid and the formation of a formaldehyde molecule. The protonation of N7 (2A-step) or N10 (2B-step) atoms are the only exergonic steps in all simulations. On the other hand, the nucleophilic attack of the water molecules on the base (1A-step) is the most endergonic step of the conversion.

We also want to highlight that SMD simulations allows us to investigate reaction mechanisms of complex systems in solution and follow the evolution of processes at molecular level, which provides information on every step about structures, and thermodynamics and kinetics properties through energy profiles.

## Author Contributions

All authors listed have made a substantial, direct and intellectual contribution to the work, and approved it for publication.

### Conflict of Interest Statement

The authors declare that the research was conducted in the absence of any commercial or financial relationships that could be construed as a potential conflict of interest.

## Supplementary Material

Table S1 The Cartesian coordinates of the initial system in solution from the electronic structure calculations.

Table S2 The Cartesian coordinates of intermediates and transition states for the different mechanisms from gas and solution SMD simulations.
