# Supplementary material for: Theoretical Study of Adenine to Guanine Transition Assisted by Water and Formic Acid Using Steered Molecular Dynamic Simulations
Source: Front Chem. 2019 Jun 12;7:414. doi: 10.3389/fchem.2019.00414 (PMC6582222; doi:10.3389/fchem.2019.00414)
Supplement: Table S1 — The Cartesian coordinates of the initial system in solution from the electronic structure calculations. [file Table_1.DOCX]

**Supporting Information**

(Total pages: 26)

**Theoretical study of adenine to guanine conversion assisted by water and formic acid using steered molecular dynamic simulations.**

S. Tolosa^*^, J.A. Sansón, A. Hidalgo

*Departamento de Ingeniería Química y Química Física,*

*Universidad de Extremadura, Badajoz, Spain.*

* Corresponding author: santi@unex.es

**Table S1**

Cartesian coordinates (in Å) obtained from reactant structures.

| **Reactant structure**  N 4.8148140 0.4342403 -3.8741160  C 6.3131250 0.1483575 -3.8741160  H 6.7643000 -0.832887 -3.8741160  N 6.9881910 1.3272360 -3.8741160  C 6.1211160 2.3612190 -3.8741160  C 6.4104360 3.7223630 -3.8741160  N 7.6535160 4.1663820 -3.8741160  H 8.4233130 3.5125370 -3.8741160  H 7.8348630 5.1599680 -3.8741160  N 5.3589030 4.5644160 -3.8741160  C 4.0824460 4.1101460 -3.8741160  H 3.2728150 4.8249180 -3.8741160  N 3.7904740 2.7902030 -3.8741160  C 4.8148140 1.9367760 -3.8741160  H 4.0360530 -0.208901 -3.8741160  C 2.5095070 4.9160750 -1.8632790  O 2.5095070 6.1360750 -1.8632790  O 3.6873020 4.2360750 -1.8632790  H 1.5742000 4.3760750 -1.8632790  H 3.5118780 3.2922390 -1.8632790  O 6.3244770 4.0227290 -5.8736200  H 7.2193750 4.3701770 -5.8797940  H 6.3516070 3.0671410 -5.9614560 |
| --- |

**Table S2**

Cartesian coordinates (in Å) obtained from SMD simulations for intermediate and transition states for gas conversion process.

| **TS1 structure**  N 0.822000027 8.04699993 -0.44900000  C 0.014000000 9.11600018 -0.78200000  H 0.423999995 9.87199974 -1.42999995  N -1.22399998 9.08899975 -0.18199999  C -1.19000006 7.91499996 0.60399997  C -2.13899994 7.23199987 1.50500000  N -3.13599992 8.15400028 2.21799994  H -3.16199994 9.10900021 1.86699998  H -3.14199996 8.07400036 3.24799991  N -1.57500005 6.28599977 2.39400005  C -0.384999990 5.78399992 2.06699991  H -0.0209999997 4.96799994 2.71399999  N 0.523000002 6.15700006 1.09500003  C 0.0930000022 7.26100016 0.437999994  H 1.76900005 7.93100023 -0.647000015  C 2.50999999 4.91599989 -1.86300004  O 2.50999999 6.13600016 -1.86300004  O 3.68700004 4.23600006 -1.86300004  H 1.57400000 4.37599993 -1.86300004  H 3.51200008 3.29200006 -1.86300004  O -3.38800001 6.61899996 0.721000016  H -3.69000006 5.81899977 1.16400003  H -4.08400011 7.52299976 1.42999995 |
| --- |

| **I1 structure**  N 6.08099985 8.76700020 1.47000003  C 5.15299988 8.78999996 2.54299998  H 5.46400023 8.44900036 3.54900002  N 3.98300004 9.22099972 2.16199994  C 4.06899977 9.54599953 0.806999981  C 3.03200006 9.96100044 -0.150000006  N 1.91799998 8.63500023 -0.179000005  H 2.18899989 7.94700003 0.551999986  H 1.77300000 8.25500011 -1.11000001  N 3.49399996 10.1770000 -1.45500004  C 4.80100012 9.95199966 -1.76300001  H 5.04600000 10.1590004 -2.82699990  N 5.75099993 9.41399956 -0.976000011  C 5.38800001 9.24300003 0.328999996  H 7.03700018 8.59099960 1.43499994  C 2.50999999 4.91599989 -1.86300004  O 2.50999999 6.13600016 -1.86300004  O 3.68700004 4.23600006 -1.86300004  H 1.57400000 4.37599993 -1.86300004  H 3.51200008 3.29200006 -1.86300004  O 2.08200002 10.8190002 0.381999999  H 1.69599998 11.4340000 -0.301999986  H 1.09899998 9.08600044 0.151999995 |
| --- |

| **TS2 structure**  N 9.43799973 8.12100029 -1.37300003  C 9.48900032 6.71700001 -1.08000004  H 10.3210001 6.05900002 -1.29600000  N 8.31700039 6.21199989 -0.583000004  C 7.52099991 7.32999992 -0.402000010  C 6.20699978 7.53000021 8.60000029E-02  N 5.24100018 6.67000008 -1.25800002  H 4.27199984 6.84899998 -1.11000001  H 5.35500002 5.67999983 -1.10699999  N 5.75400019 8.83600044 0.104000002  C 6.51000023 9.88799953 -0.296000004  H 5.96299982 10.8879995 -0.171000004  N 7.76200008 9.85400009 -0.848999977  C 8.21599960 8.54199982 -0.870000005  H 10.2209997 8.72099972 -1.41400003  C 2.50999999 4.91599989 -1.86300004  O 2.50999999 6.13600016 -1.86300004  O 3.68700004 4.23600006 -1.86300004  H 1.57400000 4.37599993 -1.86300004  H 3.51200008 3.29200006 -1.86300004  O 5.69999981 7.04899979 1.15100002  H 5.04899979 8.35799980 1.23699999  H 5.54300022 6.81699991 -2.17799997 |
| --- |

| **I2 structure**  N 7.65100002 10.2679996 -2.02200007  C 8.02900028 10.6689997 -3.31299996  H 9.10000038 10.9289999 -3.59899998  N 7.04099989 10.6040001 -4.21500015  C 5.96400023 10.0550003 -3.50799990  C 4.69199991 9.65100002 -3.99600005  N 2.88599992 7.39400005 -6.69700003  H 2.26999998 7.68800020 -6.03100014  H 2.44000006 7.54799986 -7.61199999  N 3.88199997 9.00599957 -2.99499989  C 4.32000017 8.83699989 -1.69500005  H 3.67000008 8.36800003 -0.870999992  N 5.48999977 9.24300003 -1.14800000  C 6.34499979 9.77400017 -2.09400010  H 8.11600018 10.3820000 -1.14900005  C 2.50999999 4.91599989 -1.86300004  O 2.50999999 6.13600016 -1.86300004  O 3.68700004 4.23600006 -1.86300004  H 1.57400000 4.37599993 -1.86300004  H 3.51200008 3.29200006 -1.86300004  O 4.17199993 9.77299976 -5.12400007  H 3.00600004 8.64900017 -3.36400008  H 3.61899996 7.99599981 -6.70499992 |
| --- |

| **TS3 structure**  N 15.3490000 4.05000019 -13.7989998  C 14.8269997 5.28900003 -13.3599997  H 14.5939999 6.06400013 -14.1429996  N 14.5839996 5.28000021 -11.9969997  C 14.8079996 3.96700001 -11.6540003  C 14.6330004 3.29200006 -10.4160004  N 14.0909996 1.73599994 -6.65199995  H 13.6610003 1.90300000 -5.82999992  H 14.5039997 2.56900001 -7.08699989  N 15.1230001 1.97000003 -10.3479996  C 15.5660000 1.25999999 -11.4440002  H 16.1790009 0.314999998 -11.2419996  N 15.6780005 1.87100005 -12.6890001  C 15.3400002 3.20600009 -12.7130003  H 15.6590004 3.76799989 -14.7040005  C 12.8730001 0.472000003 -11.9320002  O 11.9940004 0.296999991 -12.7959995  O 14.1420002 0.535000026 -12.2539997  H 12.6379995 0.572000027 -10.8129997  H 14.6789999 1.22500002 -13.2100000  O 14.0769997 3.67300010 -9.35200024  H 13.4820004 1.36099994 -7.38199997  H 15.1479998 1.50100005 -9.45800018 |
| --- |

| **I3 structure**    N 20.6609993 5.29300022 -6.42100000  C 19.9750004 6.49599981 -6.72499990  H 20.5209999 7.48400021 -6.90399981  N 18.6539993 6.33199978 -6.90799999  C 18.4410000 4.95499992 -6.63700008  C 17.1529999 4.29099989 -6.40799999  N 14.0909996 1.73599994 -6.65199995  H 13.6610003 1.90300000 -5.82999992  H 14.5039997 2.56900001 -7.08699989  N 17.2670002 3.03200006 -5.79199982  C 18.5230007 2.30399990 -5.82200003  H 18.5510006 1.56599998 -4.97100019  N 19.7900009 2.99099994 -5.75799990  C 19.6350002 4.30399990 -6.22700024  H 21.5869999 5.22900009 -6.16599989  C 17.9039993 0.418000013 -7.14099979  O 18.1949997 -0.252999991 -8.13899994  O 18.5970001 1.56200004 -7.05600023  H 17.1800003 0.193000004 -6.41099977  H 20.4689999 2.52099991 -6.34399986  O 16.0470009 4.66800022 -6.79699993  H 13.4949999 1.31599998 -7.38500023  H 16.4349995 2.48200011 -5.68900013 |
| --- |

| **TS4 structure**  N 11.8109999 -1.18299997 -0.330000013  C 10.5430002 -0.898000002 -0.976000011  H 9.61600018 -1.05499995 -0.485000014  N 10.7349997 -0.446999997 -2.21499991  C 12.0719995 -0.409999996 -2.46499991  C 12.7900000 0.101000004 -3.64499998  N 14.0909996 1.73599994 -6.65199995  H 13.6610003 1.90300000 -5.82999992  H 14.5039997 2.56900001 -7.08699989  N 14.1899996 -2.50000004E-02 -3.56599998  C 14.8920002 -0.629999995 -2.45900011  H 14.9350004 -2.01699996 -2.92000008  N 14.1420002 -0.958999991 -1.30100000  C 12.7700005 -0.815999985 -1.28699994  H 11.8990002 -1.10500002 0.652999997  C 16.3880005 -2.34200001 -3.22700000  O 16.9300003 -3.09500003 -2.44700003  O 16.1709995 -0.526000023 -2.42100000  H 16.7110004 -1.92100000 -4.15000010  H 14.6669998 -1.04299998 -0.446000010  O 12.3540001 0.465000004 -4.73500013  H 13.4949999 1.31599998 -7.38500023  H 14.7349997 0.365999997 -4.32000017 |
| --- |

| **I4 structure**  N 11.9799995 5.99399996 -12.3039999  C 12.2559996 6.47900009 -10.9779997  H 12.3400002 7.56899977 -10.7690001  N 12.4630003 5.43900013 -10.1709995  C 12.3079996 4.27699995 -10.9320002  C 12.5349998 2.90300012 -10.5730000  N 14.0909996 1.73599994 -6.65199995  H 13.6610003 1.90300000 -5.82999992  H 14.5039997 2.56900001 -7.08699989  N 12.3739996 1.97500002 -11.6569996  C 12.0179996 2.33599997 -12.9359999  H 10.4209995 2.43499994 -15.5229998  N 11.8079996 3.69700003 -13.2749996  C 11.9940004 4.60799980 -12.2700005  H 11.7550001 6.50099993 -13.1300001  C 10.8719997 2.48000002 -16.4769993  O 10.9870005 3.50900006 -17.1110001  O 11.8039999 1.47200000 -13.8170004  H 11.2790003 1.47200000 -16.8820000  H 11.7460003 3.98099995 -14.2320004  O 12.8859997 2.44499993 -9.50399971  H 13.4949999 1.31599998 -7.38500023  H 12.3579998 1.02199996 -11.4099998 |
| --- |

| **TS5 structure**  N 30.2929993 -4.07600021 -12.8870001  C 29.7430000 -3.48000002 -14.0959997  H 29.5090008 -4.12300014 -14.9429998  N 29.5319996 -2.16100001 -14.0500002  C 29.9669991 -1.77900004 -12.7709999  C 30.0799999 -0.493999988 -12.2119999  N 28.3379993 -1.72399998 -9.99300003  H 28.2169991 -1.29700005 -10.8380003  H 28.2049999 -1.19799995 -9.19499969  N 30.4470005 -0.411000013 -10.8149996  C 30.5809994 -1.57000005 -10.1079998  H 21.9650002 -0.574999988 -15.5990000  N 30.7019997 -2.83500004 -10.7180004  C 30.3610001 -2.97499990 -12.0209999  H 30.2210007 -4.98000002 -12.5270004  C 22.9169998 -0.218999997 -15.2189999  O 22.9790001 0.681999981 -14.3520002  O 30.9370003 -1.80599999 -8.89299965  H 23.8090000 -0.794000030 -15.4370003  H 31.0930004 -3.10800004 -9.41199970  O 29.7609997 0.514999986 -12.8199997  H 27.7570000 -2.57500005 -10.0389996  H 30.6599998 0.423999995 -10.3850002 |
| --- |

| **I5 structure**  N 29.5680008 -6.16400003 -9.80799961  C 29.5340004 -6.89400005 -11.0660000  H 29.2360001 -7.94299984 -11.0950003  N 29.8269997 -6.14599991 -12.1420002  C 30.1580009 -4.90500021 -11.6190004  C 30.3530006 -3.68099999 -12.3620005  N 28.3379993 -1.72399998 -9.99300003  H 28.2169991 -1.29700005 -10.8380003  H 28.2049999 -1.19799995 -9.19499969  N 30.4379997 -2.47700000 -11.5330000  C 30.3509998 -2.61599994 -10.1470003  H 21.9650002 -0.574999988 -15.5990000  N 30.1329994 -3.72300005 -9.39400005  C 29.9950008 -4.89599991 -10.1850004  H 29.6100006 -6.53299999 -8.91300011  C 22.9169998 -0.218999997 -15.2189999  O 22.9790001 0.681999981 -14.3520002  O 30.9279995 -1.45899999 -9.61699963  H 23.8090000 -0.794000030 -15.4370003  H 31.0270004 -1.63699996 -8.64200020  O 30.4300003 -3.59800005 -13.6009998  H 27.7570000 -2.57500005 -10.0389996  H 30.3390007 -1.56500006 -11.9069996 |
| --- |

| **TS6 structure**  N 29.5319996 4.28499985 -12.6759996  C 30.7810001 3.96199989 -12.0279999  H 31.5760002 3.36199999 -12.5290003  N 30.7810001 4.23400021 -10.7240000  C 29.5540009 4.88199997 -10.5030003  C 28.9610004 5.34499979 -9.26900005  N 25.3409996 5.56099987 -10.4619999  H 24.8309994 5.68699980 -11.3299999  H 24.9899998 6.32700014 -9.96100044  N 27.5849991 5.75500011 -9.48499966  C 26.9090004 5.69700003 -10.6940002  H 21.9650002 -0.574999988 -15.5990000  N 27.4500008 5.51499987 -11.8789997  C 28.7709999 5.04199982 -11.7399998  H 29.3059998 4.24800014 -13.6450005  C 22.9169998 -0.218999997 -15.2189999  O 22.9790001 0.681999981 -14.3520002  O 24.2689991 3.57100010 -9.44499969  H 23.8090000 -0.794000030 -15.4370003  H 24.3729992 2.90599990 -10.1379995  O 29.3810005 5.43699980 -8.13099957  H 24.8810005 4.51599979 -9.86200047  H 27.0060005 5.86299992 -8.64500046 |
| --- |

| **Product**  N 26.5160007 8.12199974 -8.89999962  C 25.5520000 9.08899975 -9.36400032  H 24.7409992 9.52499962 -8.75399971  N 25.8150005 9.55200005 -10.5930004  C 26.8500004 8.73999977 -11.0190001  C 27.3610001 8.73600006 -12.3649998  N 30.0070000 6.27199984 -11.8100004  H 30.3299999 6.15399981 -12.7810001  H 30.2910004 5.44500017 -11.3800001  N 28.3330002 7.77799988 -12.5620003  C 28.7830009 6.89599991 -11.5310001  H 21.9650002 -0.574999988 -15.5990000  N 28.2530003 6.82900000 -10.3020000  C 27.3129997 7.84200001 -9.99400043  H 26.4370003 7.61999989 -8.06000042  C 22.9169998 -0.218999997 -15.2189999  O 22.9790001 0.681999981 -14.3520002  O 33.0260010 6.40799999 -10.9899998  H 23.8090000 -0.794000030 -15.4370003  H 33.7270012 7.03299999 -10.8610001  O 27.0879993 9.53299999 -13.2959995  H 32.1850014 6.84299994 -10.7670002  H 28.7579994 7.81099987 -13.5509996 |
| --- |

Cartesian coordinates (in Å) obtained from SMD simulations for intermediate and transition states for solution conversion process.

| **TS1 structure**  N 26.4899998 21.2329998 12.6110001  C 27.7549992 21.8589993 12.7919998  H 28.6749992 21.4990005 12.2779999  N 27.8700008 22.7590008 13.7880001  C 26.5349998 22.7010002 14.3649998  C 25.9549999 23.6520004 15.2779999  N 27.0020008 24.0000000 16.3360004  H 27.9370003 23.6420002 16.1830006  H 26.6410007 24.1490002 17.3080006  N 24.6149998 23.4459991 15.5649996  C 24.0119991 22.5790005 14.7519999  H 22.9029999 22.5510006 15.0740004  N 24.3570004 21.6350002 13.8319998  C 25.6450005 21.7989998 13.6169996  H 26.2950001 20.6089993 11.8900003  C 22.9790001 25.2169991 18.6720009  O 22.9790001 26.4370003 18.6720009  O 24.1569996 24.5370007 18.6720009  H 22.0440006 24.6770000 18.6720009  H 23.9820004 23.5930004 18.6720009  O 26.1770000 25.2150002 14.7519999  H 25.5480003 25.9580002 15.0200005  H 27.0440006 25.1469994 15.5810003 |
| --- |

| **I1 structure**  N 29.5869999 21.0629997 16.4519997  C 30.9340000 21.0559998 16.0450001  H 31.5730000 20.1060009 16.3770008  N 31.2989998 22.2859993 15.5279999  C 30.1709995 23.1119995 15.6569996  C 29.9370003 24.6040001 15.4049997  N 30.8600006 25.5440006 16.4960003  H 31.5650005 24.9810009 16.9480000  H 30.1299992 25.9969997 16.9930000  N 28.6280003 25.0000000 15.5070000  C 27.6770000 24.1739998 15.9449997  H 26.7040005 24.7159996 16.1450005  N 27.7789993 22.8680000 16.3600006  C 29.0620003 22.3540001 16.1730003  H 28.9629993 20.4720001 16.9029999  C 22.9790001 25.2169991 18.6720009  O 22.9790001 26.4370003 18.6720009  O 24.1569996 24.5370007 18.6720009  H 22.0440006 24.6770000 18.6720009  H 23.9820004 23.5930004 18.6720009  O 30.6639996 24.9979992 14.2340002  H 30.0650005 25.5990009 13.7279997  H 31.3230000 26.2339993 15.9379997 |
| --- |

| **TS2 structure**  N 29.6299992 20.9090004 16.3150005  C 31.0489998 21.0550003 16.4519997  H 31.5529995 20.4139996 17.1669998  N 31.7430000 21.7339993 15.6070004  C 30.7719994 21.9549999 14.5740004  C 30.6690006 22.8680000 13.4580002  N 31.4470005 24.4179993 14.0819998  H 32.4119987 24.2110004 14.2180004  H 30.9599991 24.4829998 15.0179996  N 29.3379993 23.2089996 13.0340004  C 28.1739998 22.7619991 13.5979996  H 27.1299992 22.6590004 13.0970001  N 28.1280003 21.8579998 14.6040001  C 29.3999996 21.6429996 15.1949997  H 28.9629993 20.4720001 16.9029999  C 22.9790001 25.2169991 18.6720009  O 22.9790001 26.4370003 18.6720009  O 24.1569996 24.5370007 18.6720009  H 22.0440006 24.6770000 18.6720009  H 23.9820004 23.5930004 18.6720009  O 31.1800003 22.7859993 12.2639999  H 29.9009991 23.2500000 11.8509998  H 31.1429996 25.1739998 13.5620003 |
| --- |

| **I2 structure**  N 29.7189999 20.6119995 16.3659992  C 30.9130001 21.1140003 16.9169998  H 30.9930000 21.5100002 17.9480000  N 31.9150009 20.8939991 16.0380001  C 31.3770008 20.2380009 14.9049997  C 32.1339989 19.7189999 13.8350000  N 32.0719986 23.0599995 11.6479998  H 33.0000000 23.5249996 11.6230001  H 31.3770008 23.6849995 11.2580004  N 31.3080006 18.9200001 12.9560003  C 29.9950008 18.6410007 13.2700005  H 29.5540009 17.6790009 12.6960001  N 29.2670002 19.1100006 14.2910004  C 29.9249992 19.9909992 15.1750002  H 28.9629993 20.4720001 16.9029999  C 22.9790001 25.2169991 18.6720009  O 22.9790001 26.4370003 18.6720009  O 24.1569996 24.5370007 18.6720009  H 22.0440006 24.6770000 18.6720009  H 23.9820004 23.5930004 18.6720009  O 33.3440018 19.7600002 13.5089998  H 31.6329994 18.3990002 12.1980000  H 31.7840004 22.6170006 12.4899998 |
| --- |

| **TS3 structure**  N 25.2849998 20.7350006 16.6609993  C 26.7830009 20.4489994 16.6609993  H 27.2339993 19.4680004 16.6609993  N 27.4580002 21.6280003 16.6609993  C 26.5550003 22.6749992 16.4720001  C 26.6919994 24.0760002 16.1959991  N 26.6930008 26.1660004 19.4950008  H 26.6490002 25.1140003 19.3449993  H 27.6690006 26.4489994 19.7450008  N 25.5650005 24.7740002 15.7469997  C 24.2719994 24.1970005 15.6709995  H 23.5869999 24.6289997 14.9169998  N 24.0569992 22.8390007 16.0879993  C 25.2460003 22.0890007 16.4080009  H 24.5100002 20.0900002 16.6770000  C 22.9449997 25.4220009 17.9580002  O 22.9580002 26.5569992 17.5559998  O 23.4230003 24.4780006 17.1189995  H 22.5709991 25.0400009 18.9330006  H 23.3339996 23.2129993 17.1250000  O 27.8129997 24.6350002 16.1860008  H 25.6180000 25.7600002 15.5129995  H 26.0370007 26.4810009 20.1159992 |
| --- |

| **I3 structure**  N 25.2849998 20.7350006 16.6609993  C 26.7830009 20.4489994 16.6609993  H 27.2339993 19.4680004 16.6609993  N 27.4580002 21.6280003 16.6609993  C 26.5090008 22.5970001 16.4850006  C 26.7579994 23.9860001 16.1779995  N 26.6930008 26.1660004 19.4950008  H 26.6490002 25.1140003 19.3449993  H 27.6690006 26.4489994 19.7450008  N 25.6499996 24.7630005 15.9639997  C 24.3500004 24.3460007 16.2269993  H 23.6009998 24.8369999 15.4589996  N 24.0049992 22.9319992 16.2649994  C 25.1749992 22.0769997 16.4839993  H 24.6009998 20.0919991 16.5219994  C 23.8950005 26.0109997 17.9890003  O 24.5270004 26.8759995 17.4160004  O 23.9120007 24.7870007 17.5230007  H 23.1919994 26.0240002 18.9090004  H 23.3250008 22.7439995 16.9740009  O 27.8719997 24.5249996 16.0610008  H 25.8869991 25.6910000 15.7089996  H 26.1569996 26.2619991 20.3190002 |
| --- |

| **TS4 structure**  N 26.4459991 19.3050003 16.4880009  C 27.6980000 19.4120007 15.7670002  H 28.2080002 18.5160007 15.3479996  N 28.0170002 20.6529999 15.4549999  C 27.0799999 21.4389992 16.0739994  C 26.9750004 22.8750000 16.2849998  N 26.6930008 26.1660004 19.4950008  H 26.6490002 25.1140003 19.3449993  H 27.6690006 26.4489994 19.7450008  N 25.8869991 23.2929993 17.0669994  C 25.0480003 22.3770008 17.7999992  H 23.8110008 22.6439991 17.2339993  N 25.0809994 20.9950008 17.5790005  C 26.1350002 20.6119995 16.7810001  H 26.1959991 18.5440006 17.0230007  C 22.9190006 23.3899994 18.1709995  O 23.0919991 24.5690002 18.1000004  O 24.7150002 22.8530006 19.0060005  H 22.4720001 22.7110004 18.8770008  H 24.5620003 20.5529995 18.3050003  O 27.8409996 23.7430000 15.9320002  H 25.5580006 24.1980000 16.9330006  H 26.1569996 26.2619991 20.3190002 |
| --- |

| **I4 structure**  N 26.1660004 19.9729996 16.5809994  C 27.3689995 20.0720005 15.7770004  H 27.9909992 19.1830006 15.6630001  N 27.5750008 21.3139992 15.3409996  C 26.4759998 22.0590000 15.8420000  C 26.0000000 23.4200001 15.4919996  N 26.6930008 26.1660004 19.4950008  H 26.6490002 25.1140003 19.3449993  H 27.6690006 26.4489994 19.7450008  N 24.7310009 23.8279991 16.0400009  C 23.9619999 22.9629993 16.8799992  H 20.8290005 23.1310005 16.7929993  N 24.3990002 21.7320004 17.1690006  C 25.5960007 21.2399998 16.6140003  H 25.9130001 19.2000008 17.2250004  C 19.8020000 23.1970005 17.3689995  O 19.6709995 23.6079998 18.5160007  O 22.8160000 23.3929996 17.2360001  H 18.9090004 22.7660007 16.8390007  H 23.8430004 21.0760002 17.7560005  O 26.6270008 24.2350006 14.7559996  H 24.3090000 24.6560001 15.8540001  H 26.1569996 26.2619991 20.3190002 |
| --- |

| **TS5 structure**  N 29.8220005 19.5769997 16.2490005  C 31.2159996 19.7409992 16.2159996  H 31.9629993 19.0100002 16.6679993  N 31.6289997 20.9480000 15.8420000  C 30.4850006 21.5860004 15.4720001  C 30.3810005 22.8069992 14.7299995  N 27.4629993 23.6189995 16.7509995  H 26.7220001 23.1399994 17.2950001  H 27.0979996 24.6100006 16.5149994  N 29.0330009 23.2479992 14.5909996  C 27.9850006 22.3920002 14.8820000  H 25.0809994 23.8850002 14.5939999  N 28.0860004 21.0879993 15.4340000  C 29.3759995 20.7520008 15.6479998  H 29.3269997 18.7390003 16.3320007  C 24.5030003 24.4890003 15.2550001  O 24.6219997 25.6980000 15.4740000  O 26.6380005 22.3710003 14.6470003  H 23.8330002 23.8369999 15.8479996  H 26.7509995 21.1580009 15.2150002  O 31.2500000 23.6070004 14.4090004  H 28.7830009 24.1529999 14.2430000  H 28.2549992 23.3740005 17.2759991 |
| --- |

| **I5 structure**  N 30.8950005 20.9610004 15.8590002  C 32.0169983 21.4309998 15.1610003  H 33.0629997 21.0760002 15.3640003  N 31.7350006 22.3619995 14.2399998  C 30.3250008 22.4209995 14.2810001  C 29.4470005 23.3120003 13.5769997  N 27.4629993 23.6189995 16.7509995  H 26.7220001 23.1399994 17.2950001  H 27.0979996 24.6100006 16.5149994  N 28.1340008 23.1720009 14.0670004  C 27.6539993 22.2460003 15.0360003  H 25.0809994 23.8850002 14.5939999  N 28.4650002 21.3939991 15.7180004  C 29.8020000 21.5730000 15.3920002  H 30.7830009 20.1429996 16.3680000  C 24.5030003 24.4890003 15.2550001  O 24.6219997 25.6980000 15.4740000  O 26.2859993 22.1130009 14.9919996  H 23.8330002 23.8369999 15.8479996  H 25.9319992 21.3040009 15.5760002  O 29.6940002 24.1210003 12.5959997  H 27.4529991 23.7590008 13.6450005  H 28.2549992 23.3740005 17.2759991 |
| --- |

| **TS6 structure**  N 31.2810001 20.7199993 17.7560005  C 32.0349998 19.9990005 16.8619995  H 33.0299988 19.6389999 17.2049999  N 31.5230007 20.0279999 15.6800003  C 30.2539997 20.6700001 15.8319998  C 29.2299995 21.0730000 14.8909998  N 27.9379997 23.9939995 16.9090004  H 28.0170002 24.4640007 16.0289993  H 28.7789993 24.2779999 17.3500004  N 28.3369999 22.0359993 15.3260002  C 28.2730007 22.3969994 16.6709995  H 25.0809994 23.8850002 14.5939999  N 29.1070004 21.9330006 17.7479992  C 30.1089993 21.2259998 17.1340008  H 31.6690006 21.1060009 18.5049992  C 24.5030003 24.4890003 15.2550001  O 24.6219997 25.6980000 15.4740000  O 26.5450001 22.0629997 17.1439991  H 23.8330002 23.8369999 15.8479996  H 26.2880001 21.8820000 18.0160007  O 29.1849995 20.7779999 13.6759996  H 27.8439999 22.5839996 14.6129999  H 26.6320000 23.3010006 17.1739998 |
| --- |

| **Product**  N 31.2180004 20.7849998 15.7189999  C 31.7159996 20.2229996 14.5159998  H 32.3339996 19.2950001 14.4180002  N 31.2280006 20.8220005 13.4580002  C 30.3010006 21.7290001 13.8760004  C 29.6819992 22.8040009 13.1910000  N 28.4960003 24.7280006 16.1359997  H 28.3080006 25.5739994 15.6029997  H 28.6200008 24.8029995 17.1490002  N 29.3360004 23.9200001 13.9519997  C 29.2479992 23.7980003 15.4340000  H 25.0809994 23.8850002 14.5939999  N 29.7280006 22.7310009 16.0839996  C 30.3330002 21.7989998 15.3490000  H 31.2250004 20.3549995 16.5990009  C 24.5030003 24.4890003 15.2550001  O 24.6219997 25.6980000 15.4740000  O 27.1399994 27.2970009 18.6070004  H 23.8330002 23.8369999 15.8479996  H 26.1469994 27.4090004 18.6189995  O 29.3640003 22.7770004 12.0000000  H 29.1620007 24.8530006 13.6899996  H 27.4169998 27.6369991 17.7059994 |
| --- |
